# Preventing Acute Malnutrition in Young Children: Improving the Evidence for Current and Future Practice

**DOI:** 10.1371/journal.pmed.1001715

**Published:** 2014-09-02

**Authors:** Marko Kerac, Andrew Seal

**Affiliations:** 1 Leonard Cheshire Disability and Inclusive Development Centre, Department of Epidemiology & Public Health, University College London, London, United Kingdom; 2 Institute for Global Health, University College London, London, United Kingdom

## Abstract

Marko Kerac and Andrew Seal comment on the study by Langendorf and colleagues about the effectiveness of different strategies to prevent malnutrition in young children and discuss the implications of this study for food programs worldwide.

*Please see later in the article for the Editors' Summary*

**Linked Research Article**

This Perspective discusses the following new study published in *PLOS Medicine*:

Langendorf C, Roederer T, de Pee S, Brown D, Doyon S, et al. (2014) Preventing Acute Malnutrition among Young Children in Crises: A Prospective Intervention Study in Niger. PLoS Med 11(9): e1001714. doi:10.1371/journal.pmed.1001714

Céline Langendorf and colleagues conducted a pragmatic intervention study in Niger to assess whether distributions of supplementary foods in addition to household support by cash transfer effectively reduced malnutrition in children aged 6 to 23 months.

Acute malnutrition (wasting and/or kwashiorkor) is a major global public health problem. Over 52 million children worldwide have wasting. 875,000 deaths per year in children aged 1–59 months are attributable to wasting; of those, 516,000 are attributable to severe wasting [1]. The need to act is clear, but the evidence base is sparse [2],[3]. Evidence is particularly lacking for prevention of acute malnutrition [4]. Therefore, the study by Langendorf and colleagues in this week's issue of *PLOS Medicine* exploring the effectiveness of different strategies to prevent malnutrition in young children is both timely and important [5].

## A Pragmatic Study in a Challenging Setting

Langendorf and colleagues divided 48 rural villages in Niger into seven groups to test seven interventions [5]. Allocation was partly random, partly pragmatic. One group received cash. Three groups received cash plus different food supplements specially designed for supplementary feeding of children. One group received supplementary food and a family food ration, and two groups received the food supplements only. The primary outcome was incidence of severe acute malnutrition (SAM) and moderate acute malnutrition (MAM). 5,395 children (615–1,054 per group) were enrolled using length as a proxy for age between 6–23 months. Key findings included:

- The lowest incidences of acute malnutrition were found in the groups receiving both supplementary food and cash.
- The highest incidence of MAM was observed in the group receiving only cash, and the highest incidence of SAM was in a group receiving only supplementary food.

Risks of malnutrition were significantly greater in several of the single-intervention groups versus the combined-intervention groups, whether the single intervention was cash or supplementary food for children.

## Why the Study Matters and Its Strengths and Limitations

Implementing any large-scale trial, let alone in an emergency setting, entails major practical, political, and other types of challenges. The achievements of the study team in successfully executing their project should be applauded. As well as being one of the first of its kind, the study has many methodological strengths, including rigorous and detailed reporting and analysis. However, there are also limitations. Whilst the number of individual children involved is impressive and the use of (some) randomization and control groups is notable, this is not a randomised controlled trial or even a cluster randomised trial. Each of the seven interventions were implemented only once in each of the seven study village groups. Follow-up duration was limited, and methodologically, the trial is more akin to an observational study: rather than proving the relative effectiveness of the various interventions, findings could equally be due to inter-site differences resulting in bias or unmeasured confounding. These limitations are acknowledged by the authors, who correctly argue that “some designs may not be possible despite their explanatory benefits.” This in no way diminishes the study's importance. Prior to this study, there were all kinds of reasons to justify cash-alone interventions, food-alone interventions, or both combined in particular contexts. All could be reasonably advocated. All still *should* be advocated. However, this paper has significantly raised the bar so that future arguments can be more scientific and focused on evidence rather than ideologies. For example, if managers of a different programme in a different setting believe that these results should not be generalised and that it is best for their programme to use either cash or food alone, that would be an entirely valid stance—but only if the case were made by carefully measuring and documenting outcomes so that they can be compared and contrasted with those presented by Langendorf and colleagues.

## Implications and Ways Forward

Numerous messages arise from this paper that will play a major role in informing and shaping future policy, practice, and research. First is that intervention “packages” tend to outperform single interventions. This observation fits with malnutrition having a complex and varied aetiology. As well as immediate causes such as illness or lack of food during a poor harvest, there may be numerous more distal underlying or contributory causes for malnutrition, such as household economic vulnerability and suboptimal caring practices. Rather than a diagnosis affecting just an individual child, malnutrition is arguably better viewed as a symptom of wider problems affecting the whole family. Unless these are recognised and addressed (e.g., with cash or food support for the family, as described in the study), real and sustained improvements are unlikely. Single interventions are limited not only because they might not address the core risk factor for a particular child, but because they might not be used as intended:

- Food packages can be carefully tailored to meet a child's nutritional requirements. However, such packages are liable to be shared (thus diluting any effect on any one individual) or sold so that families can get much-needed cash.
- Unconditional cash transfers have a unique capacity to be flexibly used so that each household can address its own particular needs, such as soap for hand washing, fuel for cooking, or food itself [6]. However, because of their fungible nature, unconditional cash transfers also have the potential to be spent in a way that reduces none of the risk factors of concern.

Combination packages help to mitigate such risks and increase the likelihood of benefit. Future work should thus explore how to optimise these packages. Clinical care for common childhood illnesses (including preventative interventions, such as malaria prophylaxis) should be considered, as that, too, may have added value. Costs are also vital to consider in future work. Though differing greatly in different settings, costs are critical when arguing for and developing a budget for any major intervention scale-ups. Greater clinical effectiveness in preventing malnutrition is always desirable, but it is cost-effectiveness that is key to long-term sustainability and appeal to funders who have to make tough decisions about spending limited budgets.

Finally, this paper is a reminder that research can and should be done in challenging settings such as Niger. Global public health problems such as acute malnutrition must be tackled. The key results presented by Langendorf and colleagues all make great empirical sense. However, it is possible for plausible results from trials such as this one to be later contradicted by methodologically stronger studies [7]. We thus hope that this paper will encourage more research in this area. Programmes initially conceived as operational designs might decide to go the extra mile and transform into more formal research, e.g., step-wedge or, even better, cluster randomised trials. Published research has played a key role in revolutionising SAM/MAM treatment [8],[9]. Hopefully, the same can happen for prevention, and this study might well be a key milestone in that endeavour.

## References

[1] Black RE, Victora CG, Walker SP, Bhutta ZA, Christian P, et al (2013) Maternal and child undernutrition and overweight in low-income and middle-income countries. Lancet 10.1016/S0140-6736(13)60937-X. 10.1016/S0140-6736(13)60937-X 23746772

[2] Lang TA, White NJ, Tran HT, Farrar JJ, Day NP, et al (2010) Clinical research in resource-limited settings: enhancing research capacity and working together to make trials less complicated. PLoS Negl Trop Dis 4: e619. 20614013 10.1371/journal.pntd.0000619 PMC2894123

[3] WHO (2013) Updates on the management of severe acute malnutrition in infants and children (Guideline). Available: http://apps.who.int/iris/bitstream/10665/95584/1/9789241506328_eng.pdf. Accessed 28 July 2014. 24649519

[4] Bhutta ZA, Das JK, Rizvi A, Gaffey MF, Walker N, et al (2013) Evidence-based interventions for improvement of maternal and child nutrition: what can be done and at what cost? Lancet 382: 427–451 doi:10.1016/S0140-6736(13)60996-4 23746776 10.1016/S0140-6736(13)60996-4

[5] Langendorf C, Roederer T, de Pee S, Brown D, Doyon S, et al (2014) Preventing acute malnutrition among young children in crises: a prospective intervention study in Niger. PLoS Med 11: e1001714. 25180584 10.1371/journal.pmed.1001714 PMC4152259

[6] Bailey S, Hedlund K (2012) The impact of cash transfers on nutrition in emergency and transitional contexts. A review of the evidence. HPG Commissioned Reports. London: Overseas Development Institute. Available: http://www.odi.org/sites/odi.org.uk/files/odi-assets/publications-opinion-files/7596.pdf. Accessed 28 July 2014.

[7] Kerac M (2014) Routine antibiotics given for uncomplicated severe acute malnutrition reduce mortality and improve nutritional recovery. Evid Based Med 19(1): e1 doi:10.1136/eb-2013-101312 Epub 2013 May 21. 23695954 10.1136/eb-2013-101312

[8] Collins S (2007) Treating severe acute malnutrition seriously. Arch Dis Child 92: 453–461. 17449529 10.1136/adc.2006.098327 PMC2083726

[9] Collins S, Sadler K, Dent N, Khara T, Guerrero S, et al (2006) Key issues in the success of community-based management of severe malnutrition. Food Nutr Bull 27: S49–82. 17076213 10.1177/15648265060273S304

